# Evaluation of the Carbohydrate Composition of Crabapple Fruit Tissues Native to Northern Asia

**DOI:** 10.3390/plants12193472

**Published:** 2023-10-04

**Authors:** Zlata Stavitskaya, Lyubov Dudareva, Alexander Rudikovskii, Larisa Garkava-Gustavsson, Elena Shabanova, Alexey Levchuk, Elena Rudikovskaya

**Affiliations:** 1 Siberian Institute of Plant Physiology and Biochemistry of the Siberian Branch of the Russian Academy of Sciences, 132, Lermontov Str., Irkutsk 664033, Russia; stavitskaya.zlata@gmail.com (Z.S.); lviss12@gmail.com (L.D.); rudikovalex@mail.ru (A.R.); 2 Department of Plant Breeding, Swedish University of Agricultural Sciences, P.O. Box 190, SE 23422 Lomma, Sweden; larisa.gustavsson@slu.se; 3 Vinogradov Institute of Geochemistry of the Siberian Branch of the Russian Academy of Sciences, 1 A Favorsky Str., Irkutsk 664033, Russia; shev@igc.irk.ru; 4 A.E. Favorsky Irkutsk Institute of Chemistry of the Siberian Branch of the Russian Academy of Sciences, 1 Favorsky Str., Irkutsk 664033, Russia; on_sam@mail.ru

**Keywords:** *Malus baccata*, *Malus chamardabanica*, *Malus mandshurica*, *Malus sachalinensis*, carbohydrates, pectins

## Abstract

A comprehensive comparative analysis of the carbohydrate composition (soluble sugars and pectins) of fruit tissues of *Malus baccata*, *Malus mandshurica*, *Malus chamardabanica*, and *Malus sachalinensis*, characteristic of the vast territory of Eastern Siberia and the Far East, was carried out. It was shown that a large part of the soluble carbohydrates of the studied species were represented by transport sugars—sorbitol and sucrose. These compounds also provided the main variability in the carbohydrate composition of fruits in the studied material. The polymers pectins and protopectins isolated from the studied fruits were highly methoxylated (up to 60–70%), and their content averaged about 6% of dry weight. The greatest length of pectin polymers was found in the fruit tissues of *M. chamardabanica* and *M. sachalinensis*. Data on elemental analysis of fractions of pectins and protopectins of all studied species showed the absence of potentially toxic concentrations of heavy metals. Of note is the rather high content of calcium in both polymer fractions of the four studied species, while its content in protopectin is significantly higher. In addition, in all cases, the presence of low-molecular-weight oligosaccharide molecules with a low-dispersed linear structure was revealed in the tissues of the fruits. It is worth noting that the high content of ascorbic acid was observed in the fruits of all studied species. In addition to being of fundamental interest, information about the carbohydrate composition of the wild *Malus* species can be useful for apple breeding when choosing sources of genes underlying useful traits.

## 1. Introduction

Carbohydrates, an important component of plant tissues, are represented by various classes, including free sugars, starch, pectins, water-soluble polysaccharides, gums and mucus, as well as hemicelluloses and cellulose. Carbohydrates are synthesized in the leaves under the influence of sunlight during photosynthesis. The sugar solution containing sugars in their transport forms, such as sucrose and sorbitol, spreads from the leaves throughout the phloem to the other parts of the plant, where sugars become actively involved in metabolism [1]. During the life cycle of a plant, sugar molecules can be converted from their transport forms to storage forms as a result of enzymatic reactions [2]. Moreover, they can polymerize into cellulose and other components of the cell wall [3], glycosidize biologically active molecules (phytohormones, flavonoids, etc.) [4], participate in the process of respiration, and become converted into other compounds depending on the plant’s needs, e.g., ascorbic acid, etc. [5]. At the same time, the composition, quantity, and ratio of various types of sugars depend on many factors: the speed of formation as a result of photosynthesis, catabolism, the presence and activity of carbohydrate metabolism enzymes, etc. [6,7,8]. These processes, in turn, are genetically determined and significantly dependent on external factors (climatic conditions, soil, mineral nutrition, etc.) [9]. The genes responsible for the biosynthesis and transport of sugars through plant tissues are conventionally combined into the SWEET group [10]. The main accumulation of carbohydrates in fruiting plants occurs in the generative organs—fruits [11], where soluble sugars are known to contribute to taste, one of the most important factors for consumers, which makes fruit marketable. 

The most important fruit crop in most temperate climate regions is apple (*Malus domestica* Borkh.). The soluble sugars in apple fruit are mainly represented by fructose, sucrose, glucose, and sorbitol [12,13,14]. Due to the fact that each of the sugars has a characteristic sweetness index, the fruit taste directly depends not only on the content but also on the combination of sugar types [12,15,16]. Recent studies have shown that the total sugar content of apples did not change significantly during domestication [12,17,18]. However, their ratio changed, which means the content of fructose and glucose decreased while the content of sucrose increased [12]. 

Another important carbohydrate component involved in the formation of consumer qualities of apples is pectin. Pectins are polysaccharides involved in the formation of the cell wall [19,20]. In higher plants, pectic polysaccharides mainly consist of D-galacturonic acid residues. The carboxyl group of each D-galacturonic acid residue can exist in different states, forming salts with ions of certain metals, most often calcium, where the atoms of di- and trivalent metals can bind several chains of polygalacturonic acid, thereby forming a water-insoluble protopectin. A salt can be simultaneously methoxylated (pectinate) and remain unmodified (pectic acid is the basis of all types of pectin substances). It can also be partially methoxylated (this form is usually called pectin). The chemical properties of pectins, such as molecular weight, degree of esterification, and the content of acetyl groups, cause different biological activities in structural pectin substances [20].

The majority of the studies on carbohydrate metabolism in *Malus* are devoted to one species, *M. domestica*, where various varieties have been studied [12,16,21]. As for the wild representatives of the genus *Malus*, which grow in larch–pine forests of different ages in the vast territory of Eastern Siberia and the Far East, the carbohydrate composition of fruit tissues of these species is poorly studied. There are only a few reports on carbohydrate composition in the fruit of the Siberian crabapple, *M. baccata* (L.) Borkh. [22,23]. In addition to being of fundamental interest, the knowledge of the characteristics of the carbohydrate composition of wild *Malus* species may be useful for their use as sources of genetic variability for apple breeding. It is a necessary step towards breeding new varieties that are resistant to adverse environmental factors, such as low temperatures, drought, and fungal diseases, and that can produce good fruit quality.

Therefore, the purpose of this work was to perform a comparative analysis of the carbohydrate composition (soluble sugars and pectins) in fruits of several species like *M. baccata*, *M. mandshurica*, *M. chamardabanica*, and *M. sachalinensis*. 

## 2. Results

### 2.1. Morphological Traits 

The size and weight of the fruits of the studied species are presented in Table 1. 

**Figure 1:**
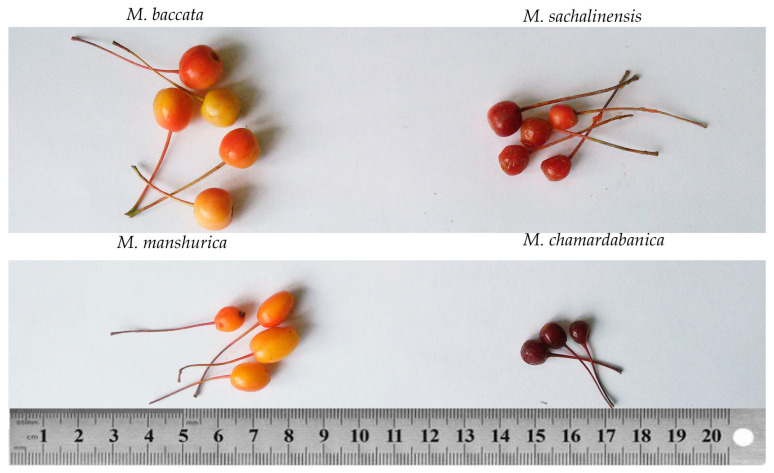
Fruits of *M. baccata*, *M. chamardabanica*, *M. mandshurica,* and *M. sachalinensis*.

The largest fruit was observed in *M. baccata*, the smallest—in *M. chamardabanica*. The fruits of *M. chamardabanica* and *M. mandshurica* had an elongated cylindrical shape (Figure 1). 

### 2.2. Content of Ascorbic Acid

A high level of ascorbic acid was noted in fruit tissues of the studied species (Table 1). The highest content was found in the *M. baccata* and the lowest—in *M. mandshurica,* while in the fruits of the other two species, the content was at about the same level. 

### 2.3. Content of the Main Soluble Sugars

**Figure 2:**
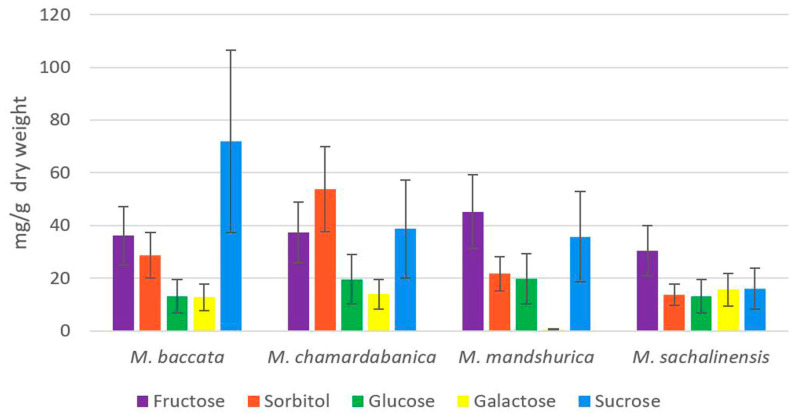
Variations in the content of soluble sugars in fruit tissues of *M. baccata*, *M. chamardabanica*, *M. mandshurica,* and *M. sachalinensis*.

**Figure 3:**
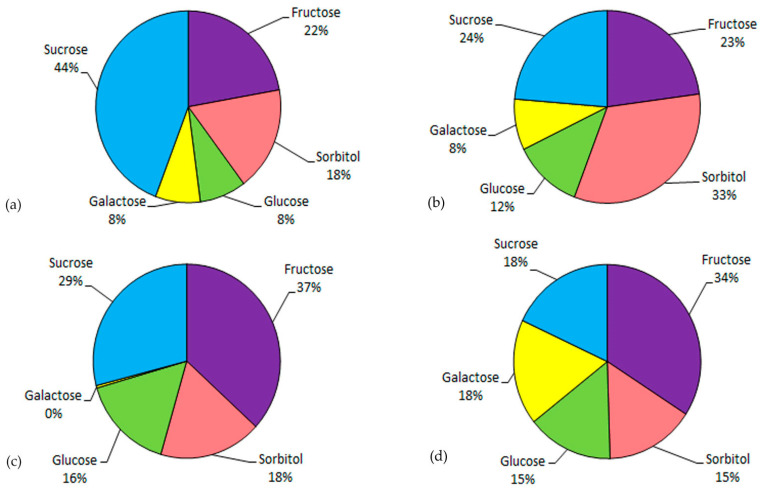
Relative variations in the content of soluble sugars in fruit tissues of (**a**) *M. baccata*, (**b**) *M. chamardabanica*, (**c**) *M. mandshurica*, and (**d**) *M. sachalinensis*.

Variations in the content of the main soluble sugars in the fruits of *M. baccata*, *M. chamardabanica*, *M. mandshurica,* and *M. sachalinensis* are presented in Figure 2 and Figure 3. 

The studied species showed species-specific patterns of distribution of the main soluble sugars. Thus, in *M. baccata*, sucrose was the dominating soluble sugar with 72 mg/g DW, while the content of other soluble sugars was considerably lower (fructose and sorbitol approximately two times; glucose and galactose approximately five times). *M. baccata* also had an approximately two-fold higher sucrose content compared to the other species. In *M. chamardabanica*, the dominating soluble sugar was sorbitol, followed by sucrose and fructose. In *M. mandshurica*, fructose and sucrose were the main sugars, while the content of galactose was detected in trace amounts. Fructose was also the dominating soluble sugar in *M. sachalinensis*, whereas the content of the four other soluble sugars was at about the same level.

Concerning the content of fructose, no significant differences were observed between the studied species. Fructose was not the main water-soluble carbohydrate in the fruit tissues of the studied *Malus* species, with the maximum observed content found in the *M. mandshurica*—about 37% of the total content (Figure 2). An important characteristic of the carbohydrate composition of the fruit tissues of the studied species was the high content of sorbitol, especially in the *M. chamardabanica,* about 33%, and about 15–18% in other species. A high content of sucrose relative to other species was also noted, up to 44% in the fruits of *M. baccata*. The fruits of the *M. sachalinensis* differed from other analyzed species by a lower (almost two times) total content of soluble carbohydrates.

### 2.4. Content of Pectin and Protopectin Fractions

Differences in the content of water-soluble pectin (WSP) and protopectin (PP) in the tissues of the fruits of the studied species are shown in Table 1. 

*M. mandshurica* is characterized by the highest content of WSP, and *M. chamardabanica* is characterized by the lowest content. The content of the PP fraction in fruits was higher than that of the water-soluble fraction, while its lowest content was also observed in the fruit of *M. chamardabanica*. No significant differences in the content of the PP fraction were observed among the other three species. In the fruit of *M. baccata, M. chamardabanica,* and *M. sachalinensis,* the content of PP was 2–2.5 times higher than that of WSP. In contrast, in the fruit of *M. mandshurica*, no significant differences were observed between the two fractions. Table 2 presents data on the average length and polydispersity of pectin and protopectin in the corresponding fractions.

It should be noted that in addition to highly polymerized pectin molecules, small linear carbohydrate oligomers were found in all the studied fractions. In the tissues of *M. baccata* fruits, the molecules, both WSP and PP, were relatively small in size (1.12 × 10^5^ and 0.99 × 10^5^ g/mol, respectively). In both fractions of the *M. chamardabanica*, in addition to the long polymer chains of WSP and PP (4.81 × 10^5^ and 4.32 × 10^5^ g/mol), shorter fragments of pectin were found. Oligomeric polysaccharides were not found in the WP fraction of the *M. mandshurica*, but the PP fraction was represented by rather long chains −3.15 × 10^5^ g/mol. The shortest polymer was found in the WSP fraction of *M. sachalinensis* fruit tissues −0.11 × 10^5^ g/mol. It is worth noting that the molecules of PP of all studied species had a much more branched structure than that of WSP. 

The degree of esterification of both WSP and PP was quite high—about 60–70% in the tissues of the fruits of all studied *Malus* species (Table 3).

### 2.5. Elemental Composition of Pectin and Protopectin Fractions 

The patterns of accumulation of micro- and trace elements in the two pectin fractions, WSP and PP, were different in different species (Table 4 and Table 5). Thus, in the WSP fraction, a relatively high accumulation of micro-elements (Ca, K, P, and S) was observed. The highest content of Ca, P, and S was found in the fruits of *M. baccata*, and the highest content of potassium was noted in the fruits of *M. chamardabanica*. On the other hand, *M. manshurica* had a lower content of Ca and K than the other species. Regarding the accumulation of microelements in the WSP, *M. baccata* demonstrated the highest content of Mn and V, *M. chamardabanica*—Cu, Fe, Zn, while the WSP from fruit tissues of *M. sachalinensis* was characterized by the absence of Cu.

Similarly, to the WSP fraction, the elements Ca, K, P, and S were also the most intensively accumulated in the PP fraction of fruit tissues of the studied species. The PP fraction in *M. baccata* showed the highest content of Ca, K, and S, while the highest sulfur content was observed in the fruits of *M. manshurica* and *M. sachalinensis*. Regarding the element composition of this fraction, *M. baccata* showed a high content of Cu, while *M. chamardabanica* did not accumulate any copper. *M. sachalinensis* was characterized by the highest content of Fe and Mn and *M. manshurica*—V and Zn. S is greater in WSP, and Ca and Fe are more actively accumulated in PP.

When comparing two pectin fractions, it can be seen that the content of potassium and phosphorus is higher in WSP and sulfur—in PP. It can also be noted that pectins of all studied species accumulate vanadium (V). Several elements like Al, Cd, Co, Ga, Li, Ni, Si, and Ti were below the limits of detection. 

### 2.6. Principal Component Analysis

Nine parameters were used for principal component analysis (PCA). The two PCs had an eigenvalue of more than 1.0 and accounted for more than 90% variation of the total biochemical variability (Table S1). PC1 was strongly associated with sucrose, which explained 62.2% of the total variability, while PC2 was associated with sorbitol and accounted for an additional 28.3% of the variability.

## 3. Discussion

### 3.1. Analysis of the Content of Main Soluble Sugars

Fruit quality attributes such as taste, aroma, and texture are of great importance for their industrial production, and the improvement of fruit quality is the most important target of apple breeding. Wild members of the genus *Malus* can be useful sources of genetic diversity in the development of new varieties. The four species involved in this study are characterized by a high level of frost tolerance and cold hardiness [24]. The use of these natural resources in apple breeding for improved cold hardiness and especially fruit quality requires more complete information about the accumulation of carbohydrates in fruits.

Our study demonstrated that fruits of all four species were characterized by a relatively low fructose content in contrast to domestic apples, where fructose and sucrose dominate [12]. These compounds also provided the main variability in the carbohydrate composition of fruits in the studied material. In crabapples growing in China [12], fructose and glucose were the main soluble sugars. Also, in *M. baccata* growing in Bulgaria [23], fructose and glucose were the main components of the complex of simple carbohydrates. In our study, glucose was not the dominant component. The highest glucose content was noted in the fruits of *M. mandshurica*, where it accounted for about 16% of all simple carbohydrates. Most of the sugars in the tissues of the fruits of *M. baccata* and *M. chamardabanica* were represented by sucrose and sorbitol. A high relative content of sorbitol was previously shown in the wild *Malus* sp. growing in China [14]. Its average content was about 5 mg/g of fruit fresh weight for cultivars and even higher, up to 16 mg/g for wild species, which is comparable with our results for the *M. baccata*. As is known, sorbitol and sucrose, the main carbohydrates found in the fruits of *M. chamardabanica* and *M. baccata*, are transport sugars that enter the generative organs through the phloem, where they are further metabolized. Most of the sorbitol is usually converted to fructose by the enzyme sorbitol dehydrogenase (SDH, EC 1.1.1.14) or into glucose by sorbitol oxidase [25]. Sucrose directly enters the fetal cell parenchyma via sucrose transporters (SUC/SUT) or is first converted to glucose and fructose via cell wall invertase (CWINV) and then transported into cells via hexose transporters [25,26]. In all likelihood, such transformations in the fruits of the studied species do not occur efficiently enough, which leads to high contents of sorbitol and sucrose and low contents of glucose and fructose in apple fruit. Li et al. [13] believe that usually, the pathways of sugar distribution function in such a way that apple fruits accumulate either a lot of sorbitol or a lot of sucrose. The species studied by us are among the smallest-fruited and, apparently, have features of the exchange and conversion of transport sugars, which results in a high content of both sorbitol and sucrose found in the fruits. The reasons for this phenomenon require a separate study.

### 3.2. Analysis of Biochemical Features of Pectin and Protopectin Fractions

The study showed that the content of pectins in the studied species is generally characteristic of the genus *Malus* (about 6% in terms of dry weight) [21]. Nevertheless, these polysaccharides had some specific characteristics. First, this was the high methoxylation of the molecules of both pectin and protopectin—60–70% [27]. It is known that the degree of esterification and, consequently, the charge of the pectin molecule is important for ensuring the optimal mechanical properties and porosity of the cell wall, which makes it possible to regulate the growth and shape of the plant cell [28].

The second feature was the presence in the tissues of the fruits of all four species of low-molecular-weight heteropolysaccharide molecules with a low dispersed linear structure, which, apparently, can be attributed to compounds such as xylogalacturonan [29] or galactan [30]. Such short-chain oligosaccharides formed during the decomposition of pectin can have physiological activity, the presence and level of which critically depends on the amount of sugar residues in the oligomer, the degree of esterification, and the availability of Ca^2+^ [31,32]. For example, in tobacco explants, they can regulate the growth and development of tissues, while their action is opposite to the effect of exogenous auxin [31], can stimulate Ca^2+^ influx into tobacco cells cultivated in suspension [33], and express the formation of proteinase inhibitors in tomato seedlings [34,35]. It has also been shown that after the addition of oligosaccharides to a suspension of soy [36] and tobacco [37], oxygen species, including H_2_O_2_ and O^2−^, can be released within a few minutes, causing an oxidative burst [38]. Active forms, which are known to induce oxygen expression, are highly pathogenic [39] and, as second messengers, induce a number of active reactions, including systemic acquired resistance and hypersensitivity reactions [40]. In addition, oligosaccharides undergo changes in cellular tissue in response to pathogenic infection [41].

As is known, the elemental composition of a plant critically depends on the properties of the soil, which provides water supply and mineral nutrition [42,43]. Thus, a plant forms a rhizosphere, which makes it possible to ensure the selectivity of the absorption of elements, which means, within certain limits, to control the chemical composition of its tissues [42,43]. Some of the mineral elements are considered biogenic and participate in plant ontogenesis, and are also capable of modulating the activity of metal-containing proteins and secondary metabolites (vitamins, flavonoids), enhancing plant resistance to stressors, fungal diseases, etc. [42]. The species we studied were grown on the same experimental site, which suggests that the differences in the accumulation of mineral substances revealed are genetically determined. It should be noted that the chemical properties of pectins ensure the formation of their complexes with various ions; therefore, the fractions of both WSP and PP are significantly enriched in minerals [44]. At the same time, their complexing ability increases with a decrease in the degree of esterification [45]. Elemental analysis data for WSP and PP fractions of all studied species showed the absence of potentially toxic concentrations of heavy metals. Attention is drawn to the rather high content of calcium in both fractions of the four species studied by us, while in PP, it is significantly higher. Calcium plays an important role in the formation of complex pectin structures [32,46]. As is known, pectins are able to form electrostatically stabilized gel networks with divalent metal cations, most often calcium [20]. Calcium may be involved in changes in cell turgor pressure and proliferation observed during fruit ripening [32]. Cross-links between calcium and pectin are a major factor in determining the physical and structural properties of fruits [32]. A high content of this element can play a significant role in reducing the extensibility of cell walls and increasing their strength [47], and the addition of a calcium chelator to the system sharply increases the extensibility of the walls [48]. It is possible that a high content of Ca^2+^ may result in the small fruit size of the studied species. In addition, according to [49], calcium content in apples correlates with resistance to several pathogens.

To date, *M. baccata* is the only species widely used in breeding from those presented in this work. A distinctive feature of its hybrids is high winter hardiness and precocity— the most important factors for agriculture. It should be noted that the other species studied in this work also have high winter hardiness. In addition, *M. chamardabanica* has a high drought tolerance, with the trees of this species characterized by low-growth habits. The *M. sachalinensis*, which grows in the humid conditions of the Far East, is resistant to scab and possibly other fungal diseases [24]. The chemical characteristics of heteropolysaccharides from the tissues of the fruits of the studied apple species could be of interest to food and pharmacological industries. The high methoxylation and significant length of these molecules can significantly affect the commercial use of pectin compounds from *M. chamardabanica* and *M. sachalinensis* as gel formers and thickeners. The small molecules of oligopolysaccharides that we discovered can have high biological activity due to their structure and can be useful in the complex therapy of dysbacteriosis as a natural prebiotic [50]. In this regard, a comparative analysis of the carbohydrate composition (soluble sugars and pectins) of fruit tissues, as the main factors that form consumer quality, is a necessary step for breeding new varieties that are resistant to adverse environmental factors: low temperatures, drought, and fungal diseases.

## 4. Materials and Methods

### 4.1. Plant Material 

The fruits of *M. baccata*, *M. mandshurica*, *M. chamardabanica,* and *M. sachalinensis* (Figure 1) were harvested from the trees grown on the same plot in the Botanical Garden of the Faculty of Biology of Moscow State University named after M.V. Lomonosov (55°42′27″ N, 37°31′38″ E). The botanical characteristics of these species are described in [24]. Fruit characteristics are described in Table 1. In total, 10–12 fruits harvested from two to three trees of each species were analyzed. The fruits were harvested at the stage of physiological maturity in 2017, 2018, and 2020. The fruit were cut into four pieces, and the seeds were removed. The fruit pieces were weighed and kept at −70 °C until being processed. The skin and flesh of the same fruit constituted a single biological replicate. 

### 4.2. Meteorological Data

The average values of day and night temperatures, as well as temperature minimums and maximums during the ripening period in 2017, 2018, and 2020, corresponded to the general climatological and phenological characteristics of the months typical for a fruit-growing area. These years were not marked by any temperature anomalies. Significant differences were observed in the amount of precipitation in 2020—159 mm in June and 175 mm in July. All meteorological data are presented in Table S2. 

### 4.3. Isolation and Analysis of Soluble Sugars

For extraction, the sample of 0.2–0.3 g was fixed in liquid nitrogen and ground to uniform fine powder to which 10 mL of 80% ethanol was added. The solution was quantitatively transferred into centrifuge tubes, sonicated for 10 min at a temperature of 15 °C in an ultrasonic bath (Sapphire, Russia), and centrifuged (Allegra 64R centrifuge, Beckman Coulter Life Sciences, USA) for 10 min at a temperature of 15 °C, 8000 rpm. The supernatant was transferred to round-bottomed flasks and evaporated dry on a rotary evaporator at a temperature of 55 °C. Then, the sample volume was adjusted to 1 mL with deionized water, and the samples were cleaned by solid-phase extraction on Diapak-AMINE concentrating cartridges. The cartridges were preliminarily washed with 5 mL of acetonitrile and 5 mL of deionized water sequentially [51]. After measuring the volume of the purified solution, the aliquot of 200 µL was transferred to an Eppendorf tube and mixed with 600 µL of acetonitrile (Cryochrome, Russia).

The complex of soluble sugars was studied by HPLC [15] on a Shimadzu LC-10 ATvp instrument (Japan), a SUPELCOSIL LC-NH_2_ column, 5 µm, with column dimensions 25 cm × 4.6 mm (Merk, Germany). The stationary phase was the phase-modified NH_2_, and the mobile phase was 75% acetonitrile in deionized water. The elution mode was isocratic, and the eluent flow rate was 1 mL/min at t = 25 °C. The compounds were identified using the retention times of standard samples: sucrose, fructose, sorbitol, galactose, and glucose (Sigma-Aldrich, USA), as well as by the method of additions.

### 4.4. Isolation of Pectin Fractions

A sample of plant material (5 g) was homogenized with the addition of 25 mL of 96% ethanol heated to 85 °C, then sonicated for 10 min in an ultrasonic bath (Sapphire, Russia). Then, the resulting solution was quantitatively transferred into round-bottomed flasks and heated for 20 min in a water bath with an air refrigerator. The heated mixture was centrifuged (Allegra 64R centrifuge, Beckman Coulter Life Sciences, USA) for 10 min, 8000 rpm, the supernatant was removed, 25 mL of deionized water heated to 45 °C was poured into the resulting precipitate, then reheated for 1 h in a water bath with an air refrigerator and centrifuged for 10 min at 8000 rpm. The supernatant was quantitatively transferred to measuring tubes, measuring the volume of the resulting water-soluble fraction of pectin (WSP).

To obtain the protopectin (PP) fraction, the remaining precipitate was mixed with 20 mL of 0.3 N hydrochloric acid, heated for 30 min in a boiling water bath with an air refrigerator, and re-centrifuged for 10 min at a speed of 8000 rpm. The supernatant was collected into a flask. The remaining precipitate was washed with hot water, and the volume was measured and mixed with a solution of ammonium citric acid in a ratio of 1:1 and heated again in a water bath for 30 min. Then, it was centrifuged again. The supernatant was mixed with hydrochloric acid extract and cooled to room temperature [52].

### 4.5. The Content of Pectic Substances

The content of pectin substances was determined by the carbazole method for the quantification of uronic acids [53]. Prior to that, the demethoxylation was carried out using the following method: 10 mL of the pectin extract was mixed with 10 mL of 0.05 N NaOH solution and left at room temperature for 30 min. After that, 10 mL of 0.05 N HCl was added to the solution. 

From each sample, 3 mL of the extract was transferred to a glass test tube where 3 mL of a 0.25% solution of Na_2_B_4_O_7_ in a concentrated H_2_SO_4_ solution was poured drop by drop. The tubes were thoroughly shaken and cooled on ice. Then, the test tubes with samples were placed in a boiling water bath for exactly 6 min and then cooled on ice. 100 mL of 0.2% carbazole solution was added and again put in a boiling water bath for exactly 10 min. The content of pectin substances was analyzed by spectrophotometric method on a Hitachi U-1100 at a wavelength of 535 nm. Galacturonic acid (Sigma-Aldrich, USA) was used to construct the calibration curve. 

### 4.6. The Molecular Weight (MW)

The molecular weight (MW) of the pectin chains was determined by HPLC [54] using an Agilent 1260 chromatographic system on a PL aquagel-OH MIXED-H 8 μm, 300 × 7.5 mm column. The column was calibrated with dextrans of known molecular weight from 106 to 1,258,000 g/mol. The column was placed in a thermostat and kept at 25 °C. The chromatograms were recorded by a refractometric detector. The temperature of the detector cell was 30 °C. The eluent solution was 0.1 M LiNO_3_, the flow rate was 1.0 mL/min, and the loop volume was 20 μL. The degree of polydispersity of polysaccharide macromolecules was determined as the ratio of the weighted average and the number average molecular weight, Mw/Mn [55].

### 4.7. Esterification of Pectin Chains

The degree of esterification (DE) of pectin samples was estimated by IR spectrophotometry using an FT-IR Spectrum One instrument (Perkin Elmer, USA) equipped with the Quant+ program with the possibility of quantitative analysis components in complex mixtures [56]. The spectra were recorded in transmission mode within the wavenumber range of 4000–400 cm^−1^ at a resolution of 4 cm^−1^. Since the DE is defined as the number of esterified carboxylic groups over the number of total carboxylic groups multiplied by 100, it is inferred that the ratio of the area of the band at 1730 cm^−1^, which corresponds to the number of esterified carboxylic groups, to the sum of the areas of the bands between 1730 and 1600 cm^−1^ that corresponds to the number of total carboxylic groups, should be proportional to the DE [21]:

$$DE\left(\%\right)=\frac{A_{1730}}{A_{1730}+A_{1600}}$$

To calculate the DE, a calibration curve based on pectin standards of known DE was constructed.

### 4.8. The Element Contents 

The contents of elements (Al, Ca, Cd, Cu, Fe, K, Mg, P, S, and Zn) in the WSP and PP, water–ethanol solutions prepared from apples and containing galacturonic acid (WSP—2–20 µg/mL; PP—1–30 µg/mL) were determined by inductively coupled plasma atomic emission spectrometry (ICP-AES) using an iCAP 6300 DUO spectrometer (Termo Scientific, UK). This instrument was equipped with an IsoMist Programmable Temperature Spray Chamber (Glass Expansion, Australia), a SeaSpray nebulizer, a cyclonic spray chamber, and a quartz plasma torch with a central tube injector. This device ensures the stability of the analytical signals in the analysis of organic solvents by cooling the spray chamber and maintaining a constant temperature of +5 °C. Operating conditions were as follows: the RF-power 1.15 kW; a plasma flow Ar-gas rate of 12 L/min; auxiliary Ar-gas flow rate of 0.5 L/min; and a nebulizer mixture of argon and oxygen gases flow rate of 0.7 L/min. Additional oxygen blowing reduced the effect of the organic component of the samples on the plasma stability. All analyses were carried out in triplicate. Results and determinations were obtained via the iTEVA software (Termo Scientific, Loughborough, UK).

The WSP and PP samples have different matrices, so the calibration solutions and samples were brought to one matrix composition. For this, background solutions of WSP (6% water solution of C_2_H_5_OH) and PP (0.0025 M HCl) were made. The sample solutions for the analyses were prepared in polypropylene tubes (15 mL) by diluting the initial sample (5 mL) in a mixture of acids (HNO_3_ (0.22 mL) and HCl (0.66 mL)). Afterward, the background solutions (1 mL) were added as follows: the PP background solution was added to the WSP samples, and vice versa, the WSP background solution was added to the PP samples. Mineral acids were additionally purified twice using a DST-1000/DST-4000 sub-boiling distillation system (Savillex corp., Eden Prairie, MN, USA). A mixture of background solutions of WSP (1 mL) and PP (1 mL) and mineral acids HNO_3_ (0.22 mL) and HCl (0.66 mL) were used as a blank control. The calibration solutions were prepared from stock solutions of single-element certified reference materials of sulfate ions (GSO No. 7480-98, UPCR Ltd., Sverdlovsk, Russia) and sodium ions (GSO No. 10228-2013, CRM Ltd., Kazan, Russia) and multi-elemental reference materials of MES-1 and MES-3 (RM No. 15608-2014 and No. 15616-2014, NPP Skat Ltd., Chelyabinsk, Russia) with the addition of the corresponding aliquots of the blank control. Each solution was adjusted to a total volume of 5 mL by ultrapure water (resistivity 18.2 MΩ/cm) obtained from a Milli-Q IQ7005 water purification system (Millipore, Burlington, MA, USA). Calibration ranges are presented in Table S3. 

### 4.9. Determination of Ascorbic Acid 

A sample of apple fruit tissues (0.3–0.5 g) was fixed in liquid nitrogen and ground in a pre-cooled mortar until a homogeneous powder was obtained. Then, 10 mL of 0.1% metaphosphoric acid was added (Sigma-Aldrich, Burlington, MA, USA). The resulting solution was quantitatively transferred to centrifuge tubes, sonicated at a temperature of 4 °C for 10 min in an ultrasonic bath (Sapphire, Stavropol, Russia), and centrifuged (Allegra 64R centrifuge, Beckman Coulter Life Sciences, Indianapolis, IN, USA) for 10 min at a temperature of 4 °C, 8000 rpm.

The content of ascorbic acid was analyzed by HPLC on a Milichrome A-02 instrument (Econova, Novosibirsk, Russia), column C18, 2 × 75, grain diameter 5 µm, 35 °C. Eluent—25% methanol solution in 0.1% metaphosphoric acid (Sigma-Aldrich, USA). The calibration curve was constructed using a standard sample of L-ascorbic acid (Sigma-Aldrich, USA).

### 4.10. Data Analyses

Data analyses were carried out using the Microsoft Excel package. In all cases, the mean values and their standard deviations were calculated. The significance of the differences between the variants was determined by the Student’s *t*-test (*p* ≤ 0.05). Principal component analysis was made using PAST 4.06 software.

## 5. Conclusions

The taste of apple fruit is, to a large extent, determined by soluble sugars. Comparative analysis of the carbohydrate composition of the fruit tissues of *M. baccata*, *M. mandshurica*, *M. chamardabanica,* and *M. sachalinensis* showed that transport sugars—sorbitol and sucrose constitute a large part of the soluble carbohydrates in these species. Furthermore, these two sugars provided the main variability in the carbohydrate composition of fruits in the studied material. Pectins and protopectins in all four species accounted for about 6% of dry weight and were highly methoxylated (up to 60–70%). The longest pectin polymers were found in the fruits of *M. chamardabanica* and *M. sachalinensis*. No potentially toxic concentrations of heavy metals were found in any of the studied species. At the same time, differences were observed in the preferential accumulation of some physiologically important elements between two fractions: water-soluble pectins and protopectins. The water-soluble pectins accumulated mainly potassium and phosphorus, while protopectins accumulated sulfur. Both fractions of pectins contained a large amount of calcium, the most important element for the formation of their structure, which plays a significant role in reducing the extensibility of cell walls and increasing their strength. Moreover, the presence of low-molecular oligosaccharide compounds with a low-disperse linear structure in the tissues of fruits was found in all species. Information about the characteristics of the carbohydrate composition of fruit tissues of wild species of the genus *Malus*, in addition to fundamental interest, can be useful in apple breeding when choosing sources of genes that carry beneficial traits. In this practical respect, it should be noted the high content of ascorbic acid in the fruits of all studied species. Further studies are needed to examine the inheritance of sugar content in hybrids between *M. domestica* and the analyzed apple species. 

## Figures and Tables

**Table 1:** Morphological and biochemical characteristics of fruit tissues of *M. baccata*, *M. mandshurica*, *M. chamardabanica,* and *M. sachalinensis*, mg/g dry weight.

Species	Diameter, cm	Length, cm	Weight, g	Ascorbic Acid, mg%	Water-Soluble Pectin, mg/g	Protopectin, mg/g
*M. baccata*	1.35 ± 0.28	1.28 ± 0.36	1.22 ± 0.25	154 ± 26	4.81 ± 0.42	9.33 ± 1.11
*M. chamardabanica*	0.84 ± 0.04	1.10 ± 0.09	0.38 ± 0.04	129 ± 22	1.88 ± 0.31	5.07 ± 0.71
*M. mandshurica*	0.92 ± 0.10	1.42 ± 0.26	0.67 ± 0.08	117 ± 28	8.63 ± 0.86	9.18 ± 1.03
*M. sachalinensis*	1.21 ± 0.10	1.21 ± 0.08	0.93 ± 0.08	127 ± 28	6.02 ± 0.81	10.80 ± 1.06

**Table 2:** Average length and polydispersity of water-soluble pectin and protopectin fractions of fruit tissues of *M. baccata, M. chamardabanica, M. mandshurica,* and *M. sachalinensis*.

	Water-Soluble Pectin				Protopectin			
Species	Pectin Molecules, g/mol	D	LMWHPM ^#^, g/mol	D	Pectin Molecules, g/mol	D	LMWHPM ^#^, g/mol	D
*M. baccata*	1.12 × 10^5^	2.1	6.90 × 10^3^	1.1	0.99 × 10^5^	2.8	11.00 × 10^3^	1.1
*M. chamardabanica*	4.81 × 10^5^	1.5	7.00 × 10^3^	1.1	4.32 × 10^5^	8.2	23.00 × 10^3^	1.2
*M. mandshurica*	ND	----	11.00 × 10^3^	1.1	3.15 × 10^5^	5.7	19.00 × 10^3^	1.3
*M. sachalinensis*	0.11 × 10^5^	6.5	6.80 × 10^3^	1.1	1.03 × 10^5^	4.0	7.30 × 10^3^	1.1

^#^ LMWHPM—low-molecular-weight heteropolysaccharide molecules. ND—Non-Detect.

**Table 3:** Degree of esterification of WSP and PP of fruit tissues of *M. baccata*, *M. mandshurica*, *M. chamardabanica,* and *M. sachalinensis*, %.

Species	Water-Soluble Pectin	Protopectin
*M. baccata*	66 ± 2	70 ± 6
*M. chamardabanica*	65 ± 11	61 ± 11
*M. manshurica*	68 ± 6	72 ± 3
*M. sachalinensis*	60 ± 1	66 ± 6

**Table 4:** Elemental composition of WSP of fruit tissues of *M. baccata*, *M. mandshurica*, *M. chamardabanica,* and *M. sachalinensis*, µg/g dry weight of pectin.

Vater-Soluble Pectin	Al	K	Mg	Ca	Cd	P	S	Cu	Fe	Zn
*M. baccata*	<0.15	79.52	8.17	21.01	<0.02	63.31	50.03	0.12	0.23	0.15
*M. chamardabanica*	<0.15	118.40	20.72	10.58	<0.02	36.17	24.45	0.18	0.39	0.54
*M. manshurica*	<0.15	24.88	4.21	10.34	<0.02	14.91	22.54	0.04	0.10	0.11
*M.sachalinensis*	<0.15	96.38	8.20	14.16	<0.02	28.32	22.73	ND	0.19	0.12

ND—Non-Detect.

**Table 5:** Elemental composition of PP of fruit tissues of *M. baccata*, *M. chamardabanica*, *M. mandshurica*, and *M. sachalinensis*, µg/g dry weight of protopectin.

Protopectin	Al	K	Mg	Ca	Cd	P	S	Cu	Fe	Zn
*M. baccata*	<0.15	25.25	3.23	45.10	<0.02	10.00	13.02	0.06	0.28	0.06
*M. chamardabanica*	<0.15	ND	0.48	36.98	<0.02	0.59	2.06	0.05	0.02	0.01
*M. manshurica*	<0.15	24.54	5.77	42.70	<0.02	6.20	614.92	0.04	0.33	0.13
*M.sachalinensis*	<0.15	15.39	2.63	41.34	<0.02	3.69	83.70	0.03	0.70	0.06

ND—Non-Detect.

## Data Availability

Not applicable.

## Supplementary Materials

The following supporting information can be downloaded at: https://www.mdpi.com/article/10.3390/plants12193472/s1, Table S1: Eigenvalues for principal components obtained from PCA and significant characters within each component in the studied *Malus* species (*M. baccata, M. chamardabanica, M. mandshurica, M. sachalinensis*); Table S2: Meteorological data for the entire research period, from 2015 to 2020 years; Table S3: Ranges of calibration curves.
