# Supplementary material for: Evaluation of the Carbohydrate Composition of Crabapple Fruit Tissues Native to Northern Asia
Source: Plants (Basel). 2023 Oct 4;12(19):3472. doi: 10.3390/plants12193472 (PMC10575056; doi:10.3390/plants12193472)
Supplement: Supplementary file 1 [file plants-12-03472-s001.zip › plants-2624242-supplementary.pdf]

Supplementary

Table S1. Eigenvalues for principal components obtained from PCA and significant characters within each component in the studied *Malus* species (*M. baccata, M. chamardabanica, M. mandshurica, M. sachalinensis*).

Parameter	PC 1	PC 2
Weight	0.0031846	−0.019089
Ascorbic acid	0.0048165	−0.0030743
Water-soluble pectin	−0.048718	−0.1214
Protopectin	−0.043461	−0.13232
Fructose	0.061197	0.002604
Sorbitol	0.39019	**0.8845**
Glucose	0.0027656	0.14067
Galactose	0.0092162	0.11118
Sucrose	**0.91631**	−0.39101

Table S2. Meteorological data for the entire research period, from 2015 to 2020 years.

**2015**						
Daytime temperature, °C			Nighttime temperature, °C			
Average value	Minimum	Maximum	Average value	Minimum	Maximum	Precipitations, mm
21.8	15.0	29.1	17.3	12.4	24.4	79.0
24.4	19.6	29.8	18.5	14.6	23.3	28.0
21.4	13.8	29.3	13.7	7.8	22.0	62.0
16.8	8.8	25.8	11.3	4.6	17.9	88.0
**2016**						
Daytime temperature, °C			Nighttime temperature, °C			
Average value	Minimum	Maximum	Average value	Minimum	Maximum	Precipitations, mm
21.1	7.8	31.0	14.8	6.8	23.2	61.0
24.1	15.3	29.9	17.4	12.2	20.8	122.0
22.9	13.6	31.9	16.1	7.4	20.9	167.0
16.0	11.6	21.3	12.2	9.3	15.8	59.0
**2017**						
Daytime temperature, °C			Nighttime temperature, °C			
Average value	Minimum	Maximum	Average value	Minimum	Maximum	Precipitations, mm
17.0	8.2	24.4	11.7	3.8	17.6	140
20.7	12.0	30.3	14.7	10.0	21.4	105
22.2	11.0	30.1	15.6	7.1	21.7	68
17.8	10.9	24.6	12.9	8.7	16.7	38.0
**2018**						
Daytime temperature, °C, °C			Nighttime temperature, °C, °C			
Average value	Minimum	Maximum	Average value	Minimum	Maximum	Precipitations, mm
20.7	10.9	28.7	13.6	6.2	22.1	56
23.1	16.7	29.3	17.6	12.5	21.8	93
24.2	18.0	28.9	15.2	10.3	20.4	28
21.7	18.1	24.8	13.5	9.8	17.7	78
**2019**						
Daytime temperature, °C			Nighttime temperature, °C			
Average value	Minimum	Maximum	Average value	Minimum	Maximum	Precipitations, mm
16.7	9.6	23.6	15.5	9.1	22.5	67
19.1	12.9	28.2	13.6	9.5	20.5	71
19.4	11.7	26.8	13.4	7.7	19.6	58
21.0	11.6	25.6	13.4	8.1	17.0	29
**2020**						
Daytime temperature, °C			Nighttime temperature, °C			
Average value	Minimum	Maximum	Average value	Minimum	Maximum	Precipitations, mm
22.2	9.8	29.8	15.5	8.1	21.1	159

21.7	15.3	28.6	15.7	9.7	20.1	175
20.9	13.6	28.5	14.2	9.6	19.8	34
17.7	12.4	23.4	13.4	9.4	21.5	66

Table S3. Ranges of calibration curves.

Analyte	Concentration, µg/ml	
	**min**	**max**
Al	0.3	45
Ca	0.1	45
Cd	0.001	45
Cu	0.005	45
Fe	0.05	45
K	1	20
Mg	0.005	45
P	0.3	45
Pb	0.03	40
S	0.5	380
Zn	0.001	45
